# The role of LH in follicle development: from physiology to new clinical implications

**DOI:** 10.1186/s12958-025-01353-8

**Published:** 2025-02-10

**Authors:** Maria Longo, Francesca Liuzzi, Serena De Carlini, Antonio La Marca

**Affiliations:** Department of Medical and Surgical Sciences for Children and Adults, University of Modena and Reggio Emilia, Modena, Italy

**Keywords:** Luteinizing hormone, Anti-Müllerian hormone, Antral follicle count, Folliculogenesis, Gonadotropins

## Abstract

The process of follicle development is closely regulated by two pituitary gonadotropins: follicle-stimulating hormone (FSH) and luteinizing hormone (LH). Traditionally, folliculogenesis is considered to be divided into a gonadotropin-independent phase and a gonadotropin-dependent phase. Despite this, recent evidence has demonstrated that functional LH receptors are expressed even in smaller follicles during the phase considered to be gonadotropin independent. Luteinizing hormone promotes androgen synthesis within ovarian follicles and seems to significantly contribute to accelerate and enhance the transition from the primordial to the antral stage of folliculogenesis. Thus, LH could play a fundamental role in determining the number of recruitable antral follicles, with a direct impact on the cyclic recruitment of follicles and reproductive potential. Common clinical conditions of pituitary suppression such as hypogonadotropic hypogonadism, other than pregnancy and combined oral contraceptive use, have been considered to analyze the effect of lower serum LH levels on the functional ovarian reserve. This review outlines recent findings on the mechanisms of human follicle development, based on human and animal models, with a direct focus on possible new clinical applications.

## Introduction

Throughout a woman’s reproductive lifespan, follicles are recruited and activated from the pool of quiescent follicles. The follicle development is closely regulated by follicle-stimulating hormone (FSH) and luteinizing hormone (LH), two gonadotropins secreted by the anterior pituitary gland [1]. Luteinizing hormone plays an important role in follicle growth by contributing to follicle maturation.

Follicle development consists in the activation of primordial follicles, maturing toward the primary and secondary pre-antral stage, then becoming antral follicles. During the antral stage, the majority of follicles undergo atretic degeneration. However, after reaching puberty, a subset of these follicles undergoes gonadotropin stimulation and progresses to the pre-ovulatory stage. This is followed by the selection and maturation of a single dominant follicle and ovulation [2, 3].

This folliculogenesis is usually classified into a gonadotropin-independent phase and a gonadotropin-dependent stage. During the gonadotropin-independent phase, follicle growths through the primordial, primary and secondary stages. The follicle transition from the pre-antral stage to the early antral stage, together with follicle growth and maturation, is dependent on FSH and LH [1, 3].

Recently, interest has increased in the early stages of folliculogenesis and activation of primordial follicles mechanisms, since it is known that the primordial pool of follicles is the fundamental resource on which reproductive efficiency is based. Optimizing the potential of the primordial pool could mean optimizing reproductive potential, with a consequent improvement in the reproductive chances of infertile women or women with low reproductive prognosis.

The aim of this review is to analyze the current literature data on LH role in folliculogenesis and the possible clinical implications of these findings.

### The role of LH in follicular development

Follicular development is a complex process that involves the growth and maturation of ovarian follicles. Follicles develop through primordial, primary, and secondary stages before acquiring an antral cavity. A primordial follicle consists of an immature oocyte and several surrounding somatic cells called primordial follicle granulosa cells (GCs). The increase in size, the GC proliferation, the alignment of stroma around the basal lamina, the development of an independent blood supply and the differentiation of stroma into theca externa and theca interna layers, represents the fundamental steps trough follicular development and maturation [2].

The development to the primordial follicles into pre-antral stage is known to be gonadotropin independent, even controlled by local signals originating from both the oocyte and the somatic cells [4, 5]. The LH acts on the GCs of the follicle to promote the production of growth factors such as insulin-like growth factor 1 (IGF-1) and vascular endothelial growth factor (VEGF). These growth factors promote the proliferation and differentiation of the GCs, leading to an increase in size of the follicle and to the morphological changes listed above [6].

The tonic stimulation by FSH on its receptors in the GCs guarantees antrum formation, cell division and synthesis of glycosaminoglycans, each of which are fundamental components of antral fluids [7]. The rise in FSH levels occurring during the luteal–follicular transition is a potent stimulus for follicle recruitment, and early antral follicles begin to enlarge. Moreover, FSH also stimulates estrogen production from GCs through the induction of the aromatase system that catalyzes androgen conversion into estrogens [8].

Both FSH and LH control the follicle development at the GC level, even the LH is the principal responsible of follicle remodeling [9] and plays a refined role in the last stages of folliculogenesis.

LH stimulates androgen substrate production from theca cells (TCs) transformed into estrogen by FSH-stimulated GCs. TCs remain unstimulated until the onset of puberty, when TCs androgen production resumes. Androgens are involved in the development of follicle atresia [10], contributing to the follicle selection process. Although follicle development is controlled by both gonadotropins, LH causes an orderly continuum of follicle remodeling, the expansion in overall size and the conclusion of cell division [9].

Evidence has described that the gonadotropin sensitivity of follicles starts in the earliest stages of folliculogenesis and progressively increases [11, 12]. The LH receptors (LHRs) are present from TC differentiation onward. Immunohistochemical experiments conducted using the antihuman LHR monoclonal antibody 3B5, has demonstrated that even in pre-antral follicles measuring < 1 mm in diameter, LHR is moderately expressed on TCs [13]. Other studies demonstrate the expression of immunoreactive LHR in the TCs of pre-antral and small antral follicles [11]. The identification of immunoreactive LHR on these cells suggests a role of LH in the follicular development in the earliest stages in human ovarian folliculogenesis.

**Fig. 1:**
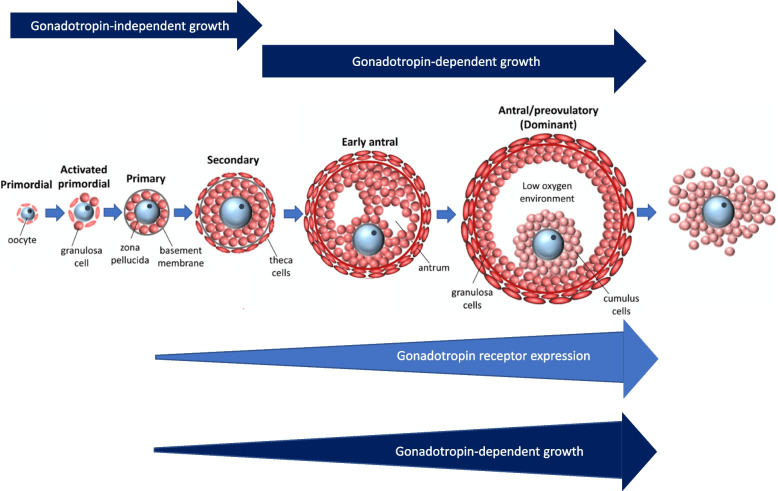
The first stage of ovarian folliculogenesis is traditionally considered to be gonadotropin independent, as indicated by the top arrows. Functional gonadotropin receptors are expressed in the granulosa cells even in the very early stages of folliculogenesis. Luteinizing hormone (LH) may exert a central role in modulating and accelerating the maturation and progression of follicles throughout folliculogenesis, increasing the rate of follicles reaching the antral stage (bottom arrow)

Pre-antral murine follicles cultured in vitro with a culture medium supplemented with human (h-)FSH and h-LH showed a critical role of LH in a dose-dependent manner [12]. Given that FSH must be continuously present, a low concentration of LH was shown to be necessary during the primary stage of follicle development to induce the FSH-dependent growth and antral development [12]. Without LH, the smaller follicles – those with one or two GC layers – do not develop beyond the large pre-antral stage, while only follicles of at least 150 μm in diameter grow through the antral stage [12]. Moreover, follicles of 85–140 μm in diameter were demonstrated to need a progressive increase in LH concentration to support the growth and antral development, with normal spindle and chromatin configuration. This observation suggests that LH might affect the development of early-stage follicles, and could be a fundamental synchronizing factor leading primary follicular growth (Fig. 1).

The importance of early timing in the addition of LH was shown to be critical since the percentage of surviving follicles was significantly higher with the addition of LH at day 6 of culture [14].

### Clinical conditions associated with relative LH deficiency

The in vitro evidence suggests a relevant role for LH in promoting and accelerating the progression of ovarian follicles throughout the different stages of folliculogenesis.

Hence, we may argue that the clinical conditions associated with absolute or relative LH deficiency may have a significant reduction into the pool of antral follicles because of a suboptimal progression of primary and small secondary follicles to the stage of recruitable antral follicles.

Clinical conditions associated to low serum LH levels are a) hypothalamic amenorrhea; b) pregnancy; c) long-term gonadotropin-releasing hormone (GnRH) analogue administration; d) hormonal contraception; e) advanced maternal age; and f) hypo-responders.

- Hypothalamic amenorrhea
  Hypothalamic amenorrhea (HA) is a pathological condition that occurs when the hypothalamic dysfunction results in a severe reduction of gonadotropin serum levels. Although women with HA have a normal ovarian reserve [15], it has been demonstrated that the chronic deficiency of GnRH and gonadotropins results in a concurrent sharp reduction of anti-Müllerian hormone (AMH) concentration, which comes close to zero [16, 17]. AMH is produced by the GCs of the small antral-stage follicles and represents a significant parameter indicating the follicular recruitment rate [15]. In case of prolonged FSH and LH deficiency, the reduced AMH levels do not represent the antral follicular count but the number of growing follicles, which is indeed diminished [16]. In relative LH deficiency, the rate of growing follicles is moderately reduced, with a moderate reduction in serum AMH levels and antral follicle count (AFC) values compared to women with gonadotropin suppression. The earlier the onset of gonadotropin deficiency, the greater the reduction in AMH values [17]. These observation leads to suppose that the first part of folliculogenesis may present some degree of gonadotropin dependency and LH could be crucial in this phase, since it promotes androgen synthesis and accelerates the rate of progression of primary follicles to the pre-antral stage.
  Evidence from a case series of women diagnosed for HA, presented for infertility with low functional ovarian reserve, demonstrate that the administration of LH (150–187.5 IU per day, or every other day) contributes to a significant increase in the functional ovarian reserve (AFC and AMH levels), indicating the supportive effect of LH on the progression of follicles throughout the early stages of folliculogenesis [18].
- Pregnancy
  There are other common clinical conditions confirming that ovarian activity is significantly controlled by gonodatropins even in the earliest phases of folliculogenesis.
  Pregnancy is marked by ovarian inactivity, associated with reduced gonadotropin concentrations. LH and FSH concentrations decrease during pregnancy, with the most significant decline occurring after the first trimester [19], returning to levels typical of a normal follicular period approximately 15–20 days after delivery [20]. This aligns with the high serum concentrations of estrogen and progesterone [20, 21], which exert negative feedback on the pituitary gland. These hormones can further diminish follicular competence to gonadotropin stimulation.
  In contrast, pregnancy is characterized by a notable increase in human chorionic gonadotropin (hCG) concentration, which binds to LHR [22]. LH, known for its short half-life and pulsatile secretion, differs from hCG, which has a longer half-life and lacks pulsatility. Since gonadotropin pulsatility is considered a crucial physiological event in ovarian folliculogenesis and steroidogenesis [23, 24], several studies [22, 25] have demonstrated that hCG and LH exhibit different activities on LHR, with hCG displaying lower proliferative potential compared to LH. The restoration of menstrual cyclicity following delivery confirms that gonadotropins play a role in promoting the progression rate of the early stages of folliculogenesis [26].
- Long-term GnRH analogue administration
  A decline in circulating levels of AMH, together with reduction in ovary volume and AFC, is significant in women treated with GnRH agonist, as well as in patients treated for early-stage breast cancer; this supports the idea that AMH production is somewhat gonadotropin sensitive [27].
- Hormonal contraception
  The use of oral contraceptive induces the quiescence of the ovary suppressing the hypothalamic-pituitary–gonadal axis with a subsequent inhibition of follicular growth [28]. Users of combined oral contraceptives (COCs) have AMH levels and AFC values significantly lower than non-hormonal contraceptive users [29]. Both ovarian reserve markers, AMH and AFC, have shown to increase and return to normal values 2 months after discontinuing use of COCs [30].
- Advanced maternal age
  Advanced maternal age (AMA) is defined as women older than 35 years. It is characterized by a decrease in the euploid rate of oocytes [31] and functional ovarian reserve, evidenced by reduced levels of AFC and AMH [32].

Aging is also associated with a progressive increase in FSH levels [33], as well as decreased bioactivity of FSH and LH. It has been demonstrated that the glycosylation of LH and FSH varies throughout the menstrual cycle and reproductive life, impacting the half-life and activity of gonadotropins [34].

In AMA, there is an increase in fully glycosylated FSH variants with lower affinity to the FSH receptor compared to variants expressed in younger women [35]. Additionally, LH forms change to less bioactive isoforms with aging, becoming more sialylated and less sulfonated [36]. The reduced action of circulating gonadotropins due to aging results in reduced steroidogenesis and decreased ovarian function. During the perimenopausal transition period, there is an increase in serum levels of gonadotropins, decreasing levels of E_2_, and a statistically significant negative correlation between luteinizing hormone/choriogonadotropin receptors (LHCGRs) and serum LH levels [37].

The decreased LH activity in aging women impairs androgen production [38, 39]; this results in much lower AMA than in younger women, negatively impacting spontaneous fertility as well as the success of IVF and the chances of live birth [31, 40].

While treatment with exogenous FSH is typically sufficient for ovarian stimulation in normo-responder women [41], combined treatment with recombinant human FSH (r-hFSH) and recombinant human LH (r-hLH) has proven more beneficial for women with FSH and LH deficiency [40, 42–45].

The benefit of combined treatment with r-hFSH and r-hLH in women of AMA is supported by a recent meta-analysis [44]. This analysis reported a higher implantation rate and clinical pregnancy rate in women treated with r-hFSH:r-hLH versus r-hFSH alone, suggesting that combined treatment may improve these outcomes for women aged 35–40 years undergoing controlled ovarian stimulation (COS) for assisted reproductive technology (ART).

Women of AMA with normal ovarian reserve are not the only group of patients who may benefit from r-hFSH and r-hLH combination treatment. Several clinical studies and meta-analyses have found that treatment with r-hFSH and r-hLH improves pregnancy outcomes in various ART populations versus r-hFSH alone, including women with a hypogonadotropic condition defined according to ICMART 2017, which refers to both gonadotropin levels and their bioactivity [46].

f) Hypo-responders
  The accurate prediction of ovarian response is essential for optimizing the management of patients undergoing COS. This prediction allows clinicians to anticipate the risk of complications. In this context, both biological and biochemical markers, such as AFC and AMH serum levels, have been considered. However, a more dynamic and interesting model for assessing hypo-responsiveness during COS is the Follicular Output Rate (FORT), introduced by Genro et al. in 2011 [47]. FORT is calculated as the ratio between the number of pre-ovulatory follicles obtained in response to COS with gonadotropin administration and the pre-existing pool of small antral follicles.

In addition to FORT, the Ovarian Sensitivity Index (OSI) was proposed by Biasoni et al. in 2011 [48]. The OSI is calculated by dividing the total administered FSH dose by the number of retrieved oocytes. It is important to note that this measure does not consider the type of gonadotropin adopted (recombinant or urinary) or the gonadotropin regimen utilized.

Recent studies demonstrate that the use of LH in women undergoing COS enhances follicle development in hypo-responder patients. This results in a better number of oocytes retrieved and a higher implantation rate compared to the administration of r-FSH alone [49]. The use of exogenous LH supplementation in COS for ART has been extensively evaluated over the past 20 years.

The pathophysiological mechanisms explaining hypo-response to gonadotropin stimulation are not completely understood and are considered a condition of hyposensitivity or ‘ovarian resistance’ to standard age- and BMI-matched doses of exogenous FSH. Several authors have postulated a link between ovarian response and individual genotype, suggesting that polymorphisms of the FSH receptor may impair ovarian response to exogenous gonadotropin [50]. The use of higher dosages of recombinant FSH has been proposed to mitigate the negative effect of FSHR polymorphisms on ovarian response, and the use of recombinant LH (r-hLH) supplementation has also been investigated to overcome hypo-responsiveness to gonadotropin stimulation [51, 52].

These investigations are based on the evidence that both FSH and LH regulate GC activity, inducing local production of inhibin B and growth factors such as insulin-like growth factors I and II (IGF-I and -II), which are important in promoting follicular maturation [53]. This suggests that LH is involved in inducing and maintaining this paracrine system of biochemical factors by acting on the theca and granulosa compartments. A recent systematic review confirms this consideration, demonstrating that the addition of rLH might be more advantageous than increasing r-FSH dosage, highlighting the crucial role of LH in follicle development, especially in specific subgroups of women, including hypo-responders [49].

### Clinical conditions associated with relative LH excess

These given are examples of pathologies with a reduction in LH values. Conversely, there are few examples of clinical cases in which the underlying pathogenetic mechanism is related to an increase in LH. One of these is certainly polycystic ovarian syndrome (PCOS), a pathological endocrinopathy of reproductive age characterized by an elevated LH/FSH ratio due to high levels of LH and reduced values of FSH [54].

In patients with PCOS, variants of LHR have been found; these LHR variants may alter pituitary LH stimulation of ovarian theca and stroma cell function, testosterone production, ovarian follicle development, LH surge–induced ovulation, and corpus luteum function, with a significant impact on reproductive pathophysiology [54].

Follicular steroidogenesis is intricately regulated by the coordinated actions of FSH and LH. FSH plays a role in upregulating the expression of CYP19A (aromatase) and LH receptors in GCs, thereby increasing their sensitivity to LH. LH promotes androgen synthesis in the TCs and enhances the expression of CYP19A in GCs [55].

Given that locally produced androgens by developing follicles facilitate the transcription of genes crucial for the transition from the reserve pool to the growth pool [56], androgen supplementation in women with low androgen concentrations has been reported to increase functional ovarian reserve [57, 58].

However, it’s worth pointing out that high concentrations of androgens also contribute to the dysfunctional formation of antral follicles [59], leading also to a pituitary-inhibiting effect.

In vivo and in vitro evidence suggesting LH receptor expression in follicles from early stages [13] and in very small pre-antral follicles [60] led to the hypothesis that elevated LH affects PCOS patients’ follicular recruitment.

In women with PCOS, the typical LH/FSH ratio is higher than the normal 1:1 ratio, and this is historically recognized as a typical aspect of PCOS pathogenesis. Insufficient FSH levels contribute to impaired follicular development, while increased LH levels enhance ovarian androgen production [61] and contribute to establishing an abnormal antral follicle pool during adolescence. As a result of raised LH/FSH ratio and several paracrine mechanisms, ovulation does not occur in patients with PCOS. These include increased AMH, which inhibits the response to FSH by GCs [62], and increased production of inhibin, which contributes itself to a relative reduction in FSH values [63, 64]. Longitudinal studies indicate an improvement in the phenotypic characteristics of PCOS as women age, evidenced by menstrual cycle regularity [65, 66] and reduced serum androgen levels [67]. This amelioration in cycle regularity may be attributed to the natural reduction of ovarian follicles and the ovarian steroid secretion.

According to the LH hypothesis, a primary neuroendocrine defect leading to exaggerated LH pulse frequency and amplitude results in ovarian hyperandrogenism and anovulation, as if the increase in LH is responsible for the onset of PCOS [68]. This theory may find validity in an interesting observation. It has been demonstrated that PCOS is a complication of women with epilepsy [69], since epileptic discharges could have a direct influence on the function of the hypothalamic-pituitary axis [70]. There are several reports in the literature suggesting an association between the administration of antiepileptic drugs (AEDs) and the development of PCOS-like symptoms [71].

Disturbance of central and/or peripheral control of hypothalamic-pituitary–gonadal axis and alteration of central neurotransmitters are all causative factors for sexual, reproductive, and gonadal abnormalities associated with epilepsy [72, 73] and epileptic discharges could have a direct influence on the function of the hypothalamic-pituitary axis [70].

However, it has been demonstrated that hepatic enzyme-inducing AEDs, such as carbamazepine and phenytoin, alter the metabolism of sex steroid hormones even if valproic acid (VPA), an enzyme inhibitor, has also been associated with a frequent occurrence of PCOS, hyperandrogenism and gonadotropin release in epileptic women [74].

In patients with PCOS variants of LH receptor have been found, allowing to assume that these variants could alter pituitary LH stimulation of ovarian theca and stroma cell testosterone production, other than ovarian follicle development. Experiments on rats demonstrate that VPA administration is associated with a decrease in number of follicles, increase in LH/FSH ratio and in atretic follicles and formation of multiple cystic follicles because of drug-related significant decrease in levels of estradiol, progesterone, FSH and LH levels [69].

These observations have also been confirmed by in vitro–cultured rat ovarian follicles, in which different VPA concentrations suppressed follicular development and aromatase expression in GCs, together with a decrease in combined levels of all steroid hormones [75].

### Possible use of recombinant LH in patients with low serum LH

The idea that androgen might regulate follicular development has traditionally been considered, despite androgen excess also enhances dysfunctional formation of antral follicles leading to PCOS phenotype. The aim of increasing the level of intraovarian levels of androgen avoiding the contraceptive effect, could be effectively reached by administering exogenous LH.

The long-lasting and profound suppression of gonadotropin secretion may be associated with low ovarian reserve markers (AMH and AFC) defining a condition of reduced ovarian reserve. The absence or the relative reduction of LH may therefore correspond in a reduction of the pool of antral follicles, allowing to hypothesize the success of a therapeutic approach assuming not a low number of primordial follicles but a slow progression of follicular growth.

Evidence from a recent case series of women diagnosed with HA demonstrates that the administration of LH contributes to a significant increase in the functional ovarian reserve (number of antral follicle and AMH levels), demonstrating the supportive effect of LH on the progression of follicles throughout the early stages of folliculogenesis [18].

## Conclusion

The reduction of AMH values and AFC during pharmacological pituitary suppression, other than in the physiological scenario as pregnancy and lactation, demonstrate that gonadotropins influence the very early stages of follicular development.

Research studies investigating the role of gonadotropin in early folliculogenesis in the human female seems to demonstrate gonadotropin sensitivity even in the earliest phases of folliculogenesis, leading to hypothesize a role in promoting the progression from primordial to pre-antral stages. These intermediate stages regulate the follicle recruitment rate, with direct implications for fertility potential, and in vitro studies report a role of LH in facilitating progression through these phases.

Some clinical conditions are recognized as representing good in vivo models for exploring the role of gonadotropin depletion on ovarian function.

Further studies are needed to better understand the precise molecular mechanism driving the LH dependence of very early stages of folliculogenesis, which class of follicles better respond to LH, and the most effective therapeutic approach for each patient. These insights could provide new treatments for infertile or subfertile women other than modulating follicular recruitment and improving follicle function, to help increase the chance of ovulation in women with a low ovarian reserve.

## Data Availability

Data sharing is not applicable to this article as no datasets were generated or analyzed during the current study.

## Abbreviations

AED: Antiepileptic drug
AFC: Antral follicle count
AMA: Advanced maternal age
AMH: Anti-Müllerian hormone
ART: Assisted reproductive technology
BMI: Body mass index
COC: Combined oral contraceptive
COS: Controlled ovarian stimulation
E_2_: Estradiol
FORT: Follicular Output Rate
FSH: Follicle-stimulating hormone
GC: Granulosa cell
GnRH: Gonadotropin-releasing hormone
HA: Hypothalamic amenorrhea
hCG: Human chorionic gonadotropin
IGF: Insulin-like growth factor
IVF: In vitro fertilization
LH: Luteinizing hormone
LHCGR: Luteinizing hormone/choriogonadotropin receptor
LHR: LH receptor
OSI: Ovarian Sensitivity Index
PCOS: Polycystic ovarian syndrome
r-FSH: Recombinant human FSH
r-LH: Recombinant human LH
TC: Theca cell
VEGF: Vascular endothelial growth factor
VPA: Valproic acid
